# A sensitive and rapid determination of zinc ion (Zn^2+^) using electrochemical sensor based on f-MWCNTs/CS/PB/AuE in drinking water

**DOI:** 10.1038/s41598-022-21926-6

**Published:** 2022-11-03

**Authors:** Gilbert Ringgit, Shafiquzzaman Siddiquee, Suryani Saallah, Mohammad Tamrin Mohamad Lal

**Affiliations:** 1 Biotechnology Research Institute, Universiti Malaysia Sabah, Jalan UMS, 88400 Kota Kinabalu, Sabah Malaysia; 2 Borneo Research Marine Institute, University Malaysia Sabah, Jalan UMS, 88400 Kota Kinabalu, Sabah Malaysia

**Keywords:** Nanoparticles, Electrochemistry

## Abstract

An electrochemical method for detecting the presence of zinc (Zn^2+^) ions in drinking water was developed using functionalized multi-walled carbon nanotubes (f-MWCNTs) and chitosan (CS). Numerous cylinder-shaped graphene molecules make up f-MWCNTs, which have a high mechanical and electrical conductivity. CS benefits from nanomaterials include biocompatibility, biodegradability, and low toxicity, which are excellent in capacity absorption of metal ions. Dangerous levels of metal ions such as zinc are currently present in drinking water as a result of human and natural activity. Zinc toxicity is associated with a variety of disorders, including Alzheimer’s, Parkinson's, diabetes, and cancer. This study incorporated f-MWCNTs and CS with Prussian blue (PB) immobilised on a gold electrode (AuE). Several parameters, including as buffers, pH, scan rate, redox indicator, accumulation time, and volume, were optimised using the cyclic voltammetry (CV) method. According to the CV method, the optimal parameters were phosphate buffered saline (0.1 M, pH 2), 5 mM Prussian blue, 200 mVs^-1^ scan rate, and 5 s accumulation time. Under ideal circumstances, the differential pulse voltammetry (DPV) method was used to determine the Zn^2+^ ions concentration range of 0.2–7.0 ppm. The limit of detection (LOD) was 2.60 × 10^–7^ mol L^-1^ with a correlation coefficient of R^2^ = 0.9777. The recovery rate of the developed sensor (f-MWCNTs/CS/PB/AuE) ranged from 95.78 to 98.96%. The developed sensor showed a variety of advantages for detecting Zn^2+^ in drinking water, including a quick setup process, quick detection, high sensitivity, and mobility. This study developed the essential sensor for monitoring Zn^2+^ levels in drinking water in the future.

## Introduction

Zinc is required in most biological activities and processes in the human body, including DNA synthesis and structural integrity, gene expression, gene transcription, immunity system function, human growth and development, reproductively heredity, incretion, neuro-transmission, cell apoptosis, cell proliferation, metalloenzyme catalysis, structural and catalytic cofactors, cellular metabolism, antioxidant enzymatic activity, oocyte maturation and fertilization^1–3^. The World Health Organization (WHO) has established that 4.58 × 10^–5^ mol L^-1^ is the safe quantity of zinc in drinking water. Japan had a safety threshold of 1.52 × 10^–5^ mol L^-1^, while Canada and the United States had a value of 7.64 × 10^–5^ mol L^-1^^6^. Zinc pollution in drinking water is increasing as a result of natural and anthropogenic activities that raise trace metal concentrations in the ecosystem. Around 27,000 metric tonnes of zinc have been released into the environment, harming ecosystems and human health^4^. The environment is getting increasingly damaging due to zinc, especially in the soil. An accumulation of zinc in one region of soil has altered its structural integrity. Despite its high zinc content, soil compositions become more saturated as it becomes more acidic. The soil becomes an unfavourable home for microorganism such as bacteria and fungi. Some bacteria may be resistant to change, whereas others will either evolve or adapt to a new habitat. As a result, microbes produced unhealthy soil, and plants grew unnaturally. When plants absorb excessive zinc, the process of photosynthesis is impacted and an enzyme necessary for plant defence is impeded^12^.

Neurotoxicological effects, such as Alzheimer's and Parkinson's disease are caused by excessive zinc consumption in the body. A zinc deficiency leads to ineffective insulin production, diarrhoea and physiological consequences^1,5^. Several analytical methods for detecting Zn^2+^ have been used including fluorescence method^1,2,7^, spectrometry method^8^, colorimetric assay^9,10^, microwave sensing method^11^, electrochemiluminescence method^12^ and chemosensor method^5,13^. When compared to electrochemical approaches, these methods have some limitations such as complex sample preparations, high costs, a well-trained operator, and a long timeframe^14–16^. The electrochemical approach has been proven to be a more promising tool for detecting Zn^2+^ because of its low cost, wide linear range, rapid analysis, portability, simplicity of instrumentation, high sensitivity, stability and selectivity^15,17–19^. Nanomaterials are widely used to enhance the performance of electrochemical sensors by detecting specific target ions at lower concentrations. Nanomaterials such as multiwalled carbon nanotubes (MWCNTs) and chitosan (CS) are used in sensing applications such as trace metal elements^20,21^, energy gap analysis^22^, hormones^23,24^ and tissues engineering^3^. Chitosan (CS) is a polymer made up of D-glucosamine and N-acetyl-d-glucosamine^26^. According to Janegitz et al.^21^, CS is the second most prevalent natural polymer after cellulose. Polysaccharide is the natural polymer found in CS^27^. This polymer is non-toxic, biocompatible, biodegradable, antimicrobial, and immunogenic (it contains functional amino and hydroxyl groups) while containing natural components^22^. Another feature is good chemical cross-linking, which improves adsorption and high selectivity for the target ion^21^. Furthermore, MWCNTs' properties may be enhanced in the presence of CS due to adhesion and compatibility capabilities^20^. MWCNTs have been widely utilised in sensing applications^25^ due to their electrical conductivity and chemical stability^26^, high electron transfer rate^20^, and chemical and biological functional groups. Prussian Blue (PB), which consists of Fe_4_[Fe(CN)_6_]^3-^, has been utilised to detect chemical contamination. PB has excellent enzymatic activity for detecting hazardous trace metal ions under acidic conditions such as Zn^2+^, which are responsible for lowering the pH of the surrounding environment^9,28^. The application of PB has been employed to detect H_2_O_2_ with high selectivity in the presence of oxygen molecules^29^. Yang et al.^30^ also reported that PB can be utilised to identify organic compounds in human serum. They discovered that it has a good electrocatalytic reaction, which enhances the performance of the electrochemical sensor. According to Alamry et al.^31^, synthesised nanomaterials are immobilised on AuE to improve surface area roughness. The CV and DPV methods were performed to examine the analyte's role in the formation of the current signal. The optimization steps were carried out using the CV method because the analyte's attachment to the surface electrode influences the development of oxidation and reduction within a certain range^32^. Meanwhile, the DPV method has been applied to analytical performance test by evaluating the formation of the surface complex and the sensitivity of the modified electrode^33^. Nanomaterials such as chitosan and functionalized multiwalled carbon nanotubes supported by PB are investigated in this study to sense Zn^2+^. Chitosan and functionalized multiwalled carbon nanotubes immobilized Prussian blue and generated good electrical conductivity under optimal conditions by detecting Zn^2+^ below the safety level. We synthesized and characterized the modified electrode by examining the formation of current signals. In the presence of Zn^2+^, we also optimized a number of parameters in the modified AuE. Zn^2+^ levels in drinking water were measured using the developed approach.

## Experimental

### Chemicals

The chemicals for preparation of the buffer (potassium hydrogen phosphate, potassium dihydrogen phosphate, sodium citrate dihydrate and sodium chloride) and zinc sulfate (ZnSO_4_) were purchased from Systerm Chemicals, Malaysia. Redox indicator chemicals such as potassium ferrocyanide (II) trihydrate and iron (III) chloride were purchased from Sigma-Aldrich (USA). Chi-tosan (CS) and multiwalled-carbon nanotubes (MWCNTs) were purchased from Sigma-Aldrich (USA). All other chemicals used were of standard reagent grade diluted using milli-Q (Millipark^®^ 40), and experiments were carried out at laboratory temperature conditions of 21.0 ± 2.0 °C.

### Instrumentations and characterizations

All electrochemical measurements were carried out using a potentiostat/galvanostat (PGSTAT) electrochemical workstation (Metrohm-Autolab B.V) with a standard three-electrodes system consisting of 5 mm diameter of bare AuE, platinum wire (Pt) and silver/silver chloride (Ag/AgCl) as the working, counter and reference electrodes, respectively (Supplement 1). Voltammograms obtained from the cyclic voltammetry (CV) and differential pulse voltammetry (DPV) were analyzed with NOVA Autolab 1.11 software. All the experiments were performed with 10 mL analytical buffer (0.1 M) in an electrochemical cell at room temperature conditions of 21.0 ± 2.0 °C. The characterization of nanomaterials was observed under scanning electron microscope (SEM) (Hitachi S-3400 N) and transmission electron microscopy (TEM) (FEI Tecnai G2 Spirit BioTWIN). The pH was measured using pH meter; Model: pH 2700 Eutech Instrument. Digital electronic microbalance (Model: TLE204E Mettler Toledo) was used to measure the weight of chemicals and reagents throughout the whole experiments.

### Functionalization of multi-walled carbon nanotubes (MWCNTs)

MWCNTs were functionalized into carboxylic group (COOH) by dissolving 1 g of MWCNTs powder dissolved with concentrated sulfuric (H_2_SO_4_) and nitric acid (HNO_3_) solution with ratio 3:1. Then, the mixture was covered with aluminum foil to prevent acid gas discharge due to the uses of strong acidic solutions and was stirred for 3 h. After 3 h, the mixture was homogenized and transferred to large volume of beaker for washing with distilled water. The functionalized MWCNTs (f-MWCNTs) were formed at a bottom of beaker, which were denser than distilled water. The large amount of distilled water used to rinse the f-MWCNTs until the pH of the water turned to pH 7. After that, the mixture was dried in the oven with temperature at 60 °C for 24 h, and then formed a f-MWCNTs powder which was readily used for sensing purposes.

### Preparation of chitosan (CS) and f-MWCNTs

A 1% of CS powder was diluted in 1% of acetic acid. The mixture stirred for 5 min and 0.08 g of f-MWCNTs was added. Briefly, CS and f-MWCNTs were prepared using ratio 1:2 by adding 0.04 g and 0.08 g, respectively into 4 mL with 1% acetic acid. Then, the mixtures were stirred for 3 h to homogenize the solution. After that, the homogenization process was conducted with sonication for 10 min. The presence of amino group from CS (NH_2_) and hydroxyl group (COOH) from f-MWCNTs undergoes self-assembled due to different charges as describe by Shalauddin et al.^26^.

### Electrode pre-treatment, preparation and electrochemical measurements

**Figure 1:**
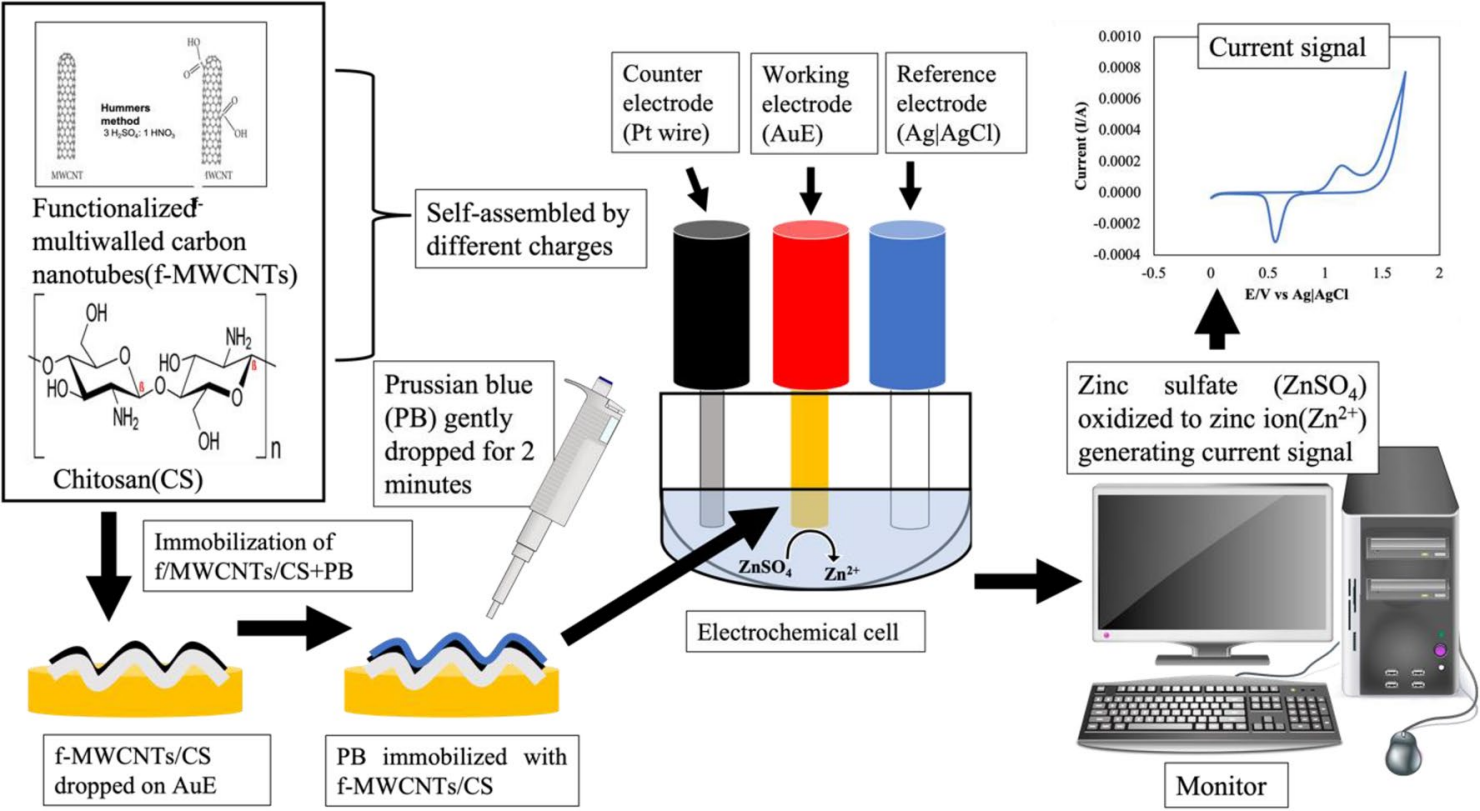
Schematic of the experiment for detection of zinc ion using electrochemical sensor.

The bare AuE pre-treatment was conducted according to the method previously described by Siddiquee et al.^34^. First, the AuE was polished with alumina slurry (0.3–0.5 µm in diameter) for two minutes followed by subsequent cleaning and rinsing with distilled water. The electrode was dried using nitrogen gas. Then, 5 µL of modification materials dropped onto the AuE surface and left it for 2 h. Then, a 10 µL solution of 5 mM Prussian blue on the surface of modified AuE (f-MWCNTs/CS/PB/AuE) was dropped for 2 min before it submerged together with the counter electrode (platinum) and reference electrode (Ag|AgCl in 3 M KCl) solution. The three-electrode systems were measured the current flows through the electrolytic solution after applying the potential current. The schematic of the work is illustrated in Fig. 1.

### Sample preparation

A sample of drinking water was collected at a local market in Kota Kinabalu, Sabah, Malaysia. Before the analysis, 2 mL of drinking water containing 0.1 ppm of manganese was diluted with (0.1 M, pH 2) PBS buffer.

## Results and discussion

**Figure 2:**
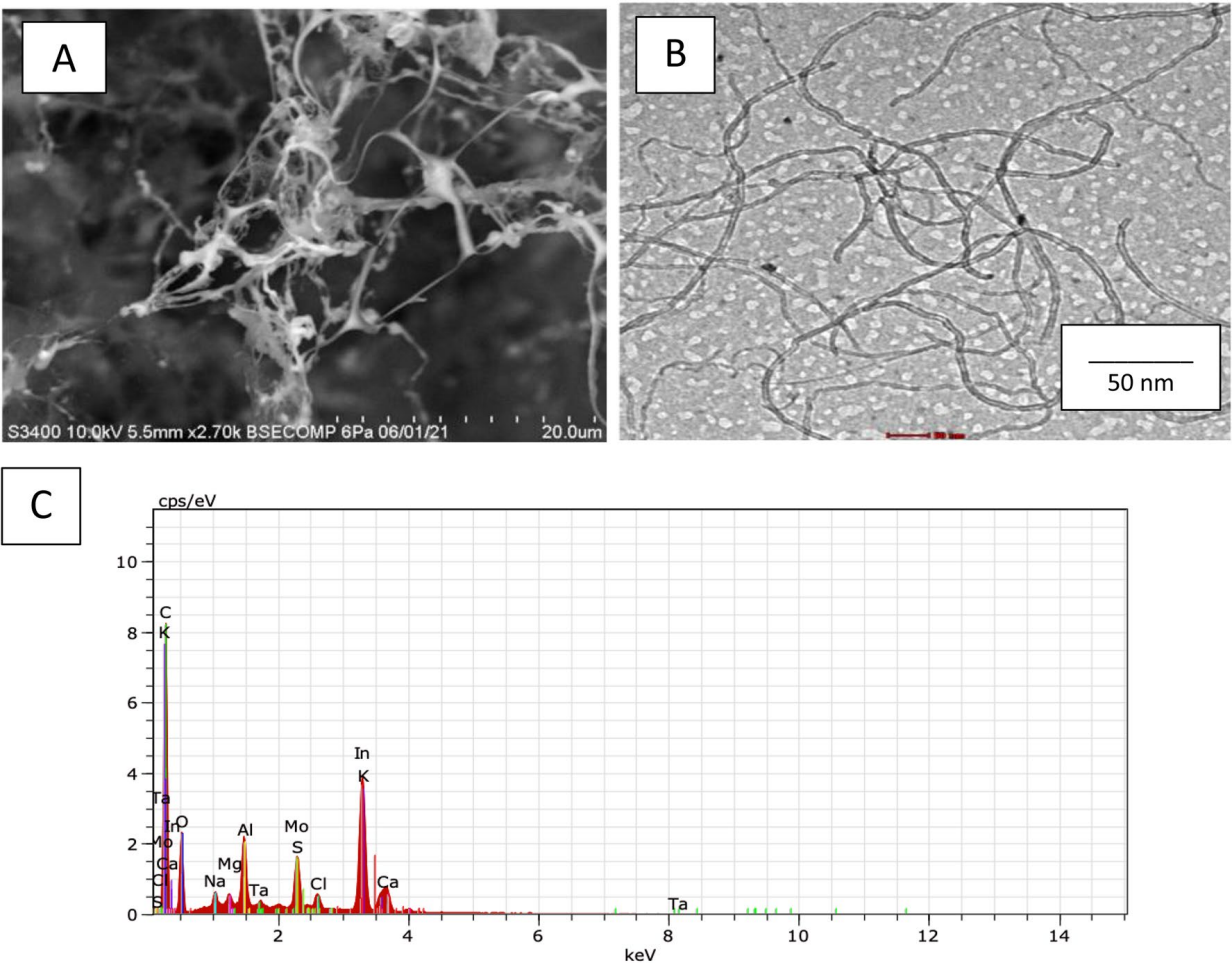
f-MWCNTs were observed under different magnifications: (**A**) SEM under magnification of 20 um, (**B**) TEM under magnification of 50 nm and (**C**) EDX analysis.

**Figure 3:**
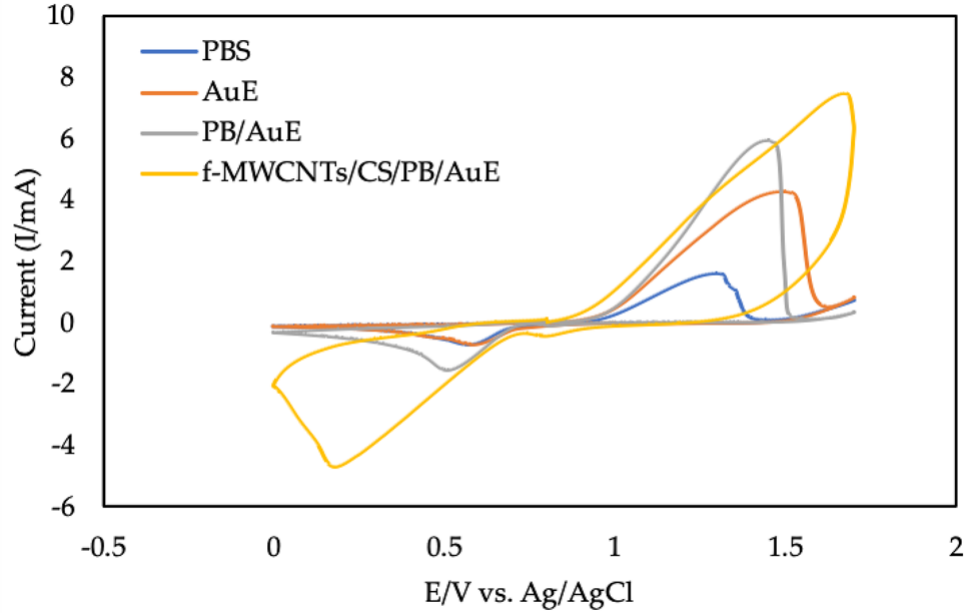
Cyclic voltammogram of electrode configurations in the presence of Zn^2+^. Experimental conditions were (0.1 M, pH 2) PBS under 250 mVs^-1^ of scan rate for 5 s accumulation time. PBS was control of the experiment in absence of Zn^2+^.

The morphological characteristics of f-MWCNTs/CS were examined under scanning electron microscope (SEM), transmission electron microscope (TEM) and energy dispersive X-ray (EDX) at Fig. 2A,B and Table 1, respectively. Figure 2A shows that when fibrous f-MWCNTs were mixed with CS, microscopic holes appeared, indicating that CS possessed a porous membrane that improved the performance of the developed sensor^3^. According to the protocol followed by Shalauddin et al.^26^, the MWCNTs were functionalized into MWCNTs-COOH, which was used to determine the specific target elements using two strong acids, sulphuric acid and nitric acid. O–H and N–H of functionalized MWCNTs (f-MWCNTs) were introduced to CS and exhibited higher current signals^26^. When coupled with CS, the f-MWCNTs were observed as well-despised pipe bundles as described by Zhao et al.^3^, as illustrated in Fig. 2B. As mentioned by the same author, these combinations were suggested to improve electron transfer rate by increasing the active surface area. Zhao et al.^3^ reported the CS and f-MWCNTs with some modifications with LOD of 2.5 × 10^–10^ mol L^-1^ for detection of diethylstilbestrol. Also, the performance of MWCNTs-chitosan was tested by Shalauddin et al.^26^ showed the LOD was 2.1 × 10^–4^ mol L^-1^ for determination of diclofenac sodium. EDX analysis was used to determine the elemental composition and percentage of f-MWCNTs/CS after synthetization step. As demonstrated in Fig. 2C, the carbon element was abundant in the f-MWCNTs/CS formations. Major elements including oxygen, aluminum, potassium, calcium, magnesium, and indium were discovered (Table 1). These elements indicated the right nanomaterials synthetization procedure. Alkene group (=CH_2_) and hydroxyl group (COOH)-containing f-MWCNTs that compact with O–H and N–H from CS^26^. The target ion's large reaction site was provided by the CS's adhesion property^3^. It is act as a natural ‘glue’ in this experiment. The electroanalytical analysis was used to see how f-MWCNTs/CS/PB affected the target ion. Then, using the CV method for detecting the formation of current signals, the influence of modified and unmodified AuE towards analyte was investigated. The CV method was used to compare bare AuE and modified AuE in the presence of Zn^2+^. Prussian blue (10 µL, 5 mM) was dropped on bare and modified AuE, resulting in modified PB/AuE and f-MWCNTs/CS/PB/AuE. Due to PB's improved biocompatibility and chemical stability as well as its lower electrical resistance, which accelerated electron transport. It was applied to both unmodified and modified AuE in this experiment. These factors merit additional study^30^. In comparison to bare AuE, both modified electrodes demonstrated the highest current signal in Fig. 3. The generation of oxidation and reduction signals was twice higher for modified AuE compared to bare AuE. Furthermore, the current indications for bare AuE were the lowest when Zn^2+^ was absent. In the presence of the target ion, f-MWCNTs/PB/CS/AuE showed high conductivity and low resistance. As a result, the highest current signals were obtained as a result of the target ion diffusion across the modified electrode’s surface area. The electrochemical behaviours of f-MWCNTs/CS/PB/AuE was further investigated on optimization experiment including buffer, pH, scan rate, accumulation duration, and volume.

**Table 1:** The elemental composition of f-MWCNTs/CS.

Element	Series (wt. %)	unn. (wt. %)	C norm. (at. %)	C. atom	(1 Sigma)
Carbon	K-series	48.24	46.35	58.98	5.53
Oxygen	K-series	37.92	36.44	34.81	4.55
Aluminium	K-series	4.58	4.40	2.49	0.24
Potassium	K-series	5.97	5.73	2.24	0.24
Calcium	K-series	1.94	1.86	0.71	0.11
Manganese	K-series	0.53	0.51	0.14	0.08
Indium	L-series	4.91	4.71	0.63	0.21
	Total	104.08	100.00	100.00

### Optimization electrochemical parameters

The initial electrochemical measurements was setting based on our previous findings using PB/AuE^35^. On a (0.1 M, pH 2) analytical solution, electrochemical measurements ranging from 0.0 V to 1.7 V were performed at a scan rate of 250 mVs^-1^ for a 5 s accumulation period.

### Effect of buffer

**Figure 4:**
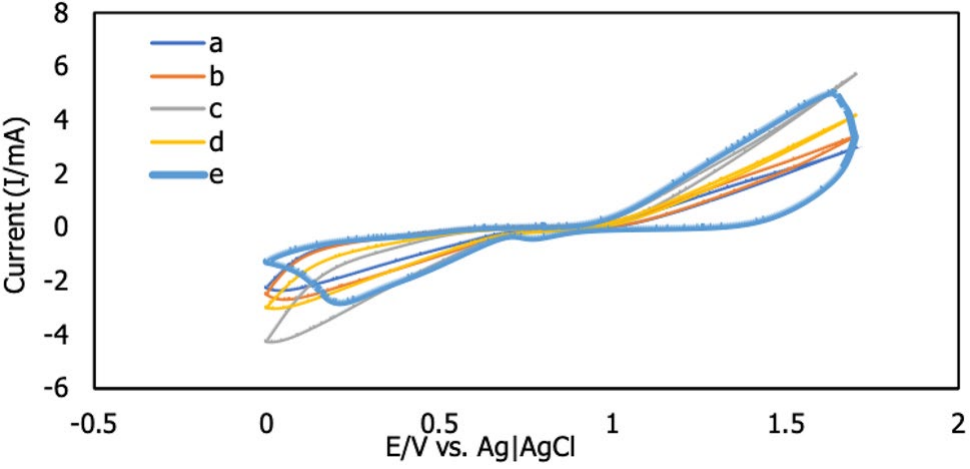
Cyclic voltammogram of different buffers tested with modified electrode (f-MWCNTs/CS/PB/AuE); (**a**) acetate (**b**) ammonium (**c**) citrate, (**d**) tris–HCl and (**e**) PBS buffers under scan rate of 250 mVs^-1^ for 5 s accumulation time in the presence of 3 ppm of Zn^2+^ (*n* = 3).

Five different buffers were investigated in the presence of Zn^2+^ ions using the CV method. At the same concentration of buffers and pH of 2, acetate buffer, phosphate-buffered saline (PBS), citrate buffer, ammonium buffer and tris–HCl buffer were prepared. Before measuring the current signals, a 10 mL of buffer containing 3 ppm Zn^2+^ was stirred for 5 min to homogenize the mixture. Figure 4 shows the effects of several types of buffers. Due to the well-shaped voltammogram graph, Zn^2+^ delivered by PBS exhibited higher current signals as oxidation and reduction, whereas the other buffers did not create any signals. The surface area of modified AuE for detecting Zn^2+^ was determined in five different buffers using the formula ip = 2.69 × 10^5^nAC_0_D^1/2^v^1/2^, which was influenced by oxidation (Ipa) and reduction (Ipc) peaks^19^. A Prussian blue transfers one electron (*n* = 1), diffusion coefficient of Prussian blue, (D = 6.67 × 10^–6^), surface-active area of f-MWCNTs/CS/PB/AuE (A), bulk concentration of redox (C_0_ = 0.005 M) and scan rate (v = 0.2 Vs^-1^). The countable surface-active areas of 0.00055 cm^2^ for Ipa and 0.0031 cm^2^ for Ipc, respectively, revealed that PBS is a good supporting solution for sensing Zn^2+^. Within a potential range of 0.0 V to 1.7 V, PBS enhanced the analyte to form oxidation and reduction signals. This result suggested that Zn^2+^ interacts electrostatically with the surface of modified AuE^36^. The magnetic field was formed by the electrostatic interaction between the zinc ion and the modified AuE surface, which drew the Zn^2+^ towards the modified AuE surface. Zn^2+^ migrated from the bulk to the modified surface of MWCNTs/CS, where it occupied the highest current signal compared to bare electrode. PBS helps in the formation of the highest current signals by the modified electrode. As a result, PBS was chosen for further evaluation.

### Effect of pH

**Figure 5:**
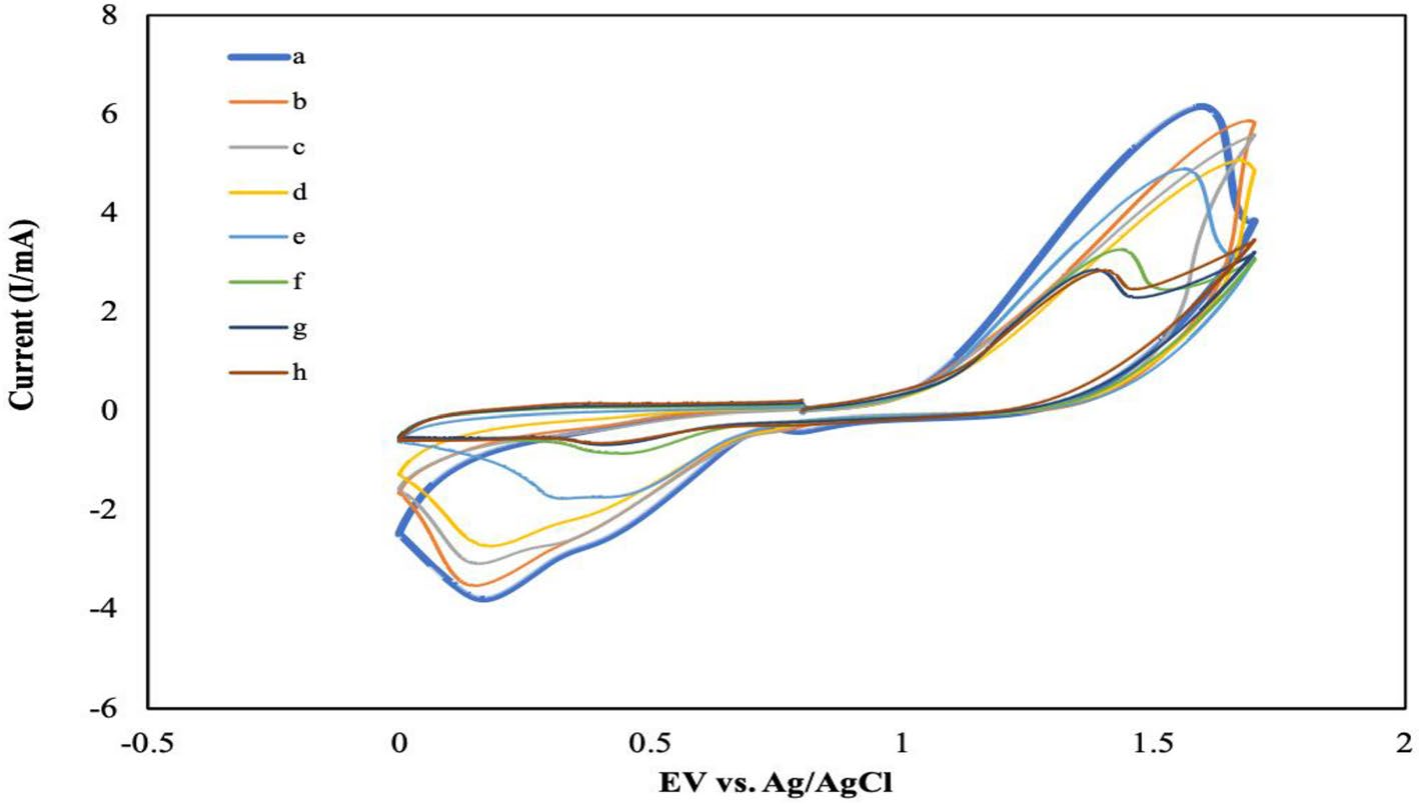
Cyclic voltammogram’s effect of different pH values tested on f-MWCNTs/CS/PB/AuE; (**a**) pH 2, (**b**) pH 3, (**c**) pH 4, (**d**) pH 5, (**e**) pH 6, (**f**) pH 7, (**g**) pH 8 and (**h**) pH 9 under scan rate of 250 mVs^-1^ for 5 s accumulation time in the presence of 3 ppm of Zn^2+^ (*n* = 3).

The influence of pH was investigated using 0.1 M PBS in the presence of 3 ppm Zn^2+^ from pH 2 to pH 9, with a pH interval of 1. Figure 5 shows the current signals in a cyclic voltammogram graph. Oxidation currents were created at potentials ranging from 0.8 to 1.7 V, while reduction currents were formed at potentials ranging from 0.0 to 0.8 V. The pH value was disproportioned in both current signals. The current signals increased as the pH of the buffer became more acidic, and they decreased as the pH of the buffer became more alkaline. For an oxidation reaction, several signals were found to be unstable. Based on the results, the current signals from pH 3–5 appeared to be shifting further towards positive potential range. According to De Oliveira et al.^37^, this phenomenon occurred due to the presence of an additional ion, hydrogen ion, H^+^. This experiment demonstrated that the acidity of the solution had an effect on the sensor's current intensity. In addition, the presence of H^+^ as a manipulator solution was used to examine the analyte signal at various pH levels. It was discovered that as the pH value decreased, the current intensity of the analyte increased. Meanwhile, Behnia et al.^38^ reported that greater pH levels than pH 5.5 could result in Zn^2+^ precipitation. The precipitation of Zn^2+^ happened when the pH value approached pH 9 (h) due to the precipitation process between pH 9.0 and 9.5. Another intriguing finding connected to the buffer's acidic condition was its stability at pH 2. In comparison to other pH values, the current signal formed the highest peak currents and stable for oxidation and reduction with a well-shaped curve at pH 2. 6.166 mA at 1.596 V was the oxidation peak, while -3.779 mA at 0.1676 V was the reduction peak. This result demonstrated that in acidic conditions, heavy metal ions such as Zn^2+^ are extremely reactive. As a result, pH 2 was chosen as the best candidate for detection of Zn^2+^.

### Effect of scan rate

**Figure 6:**
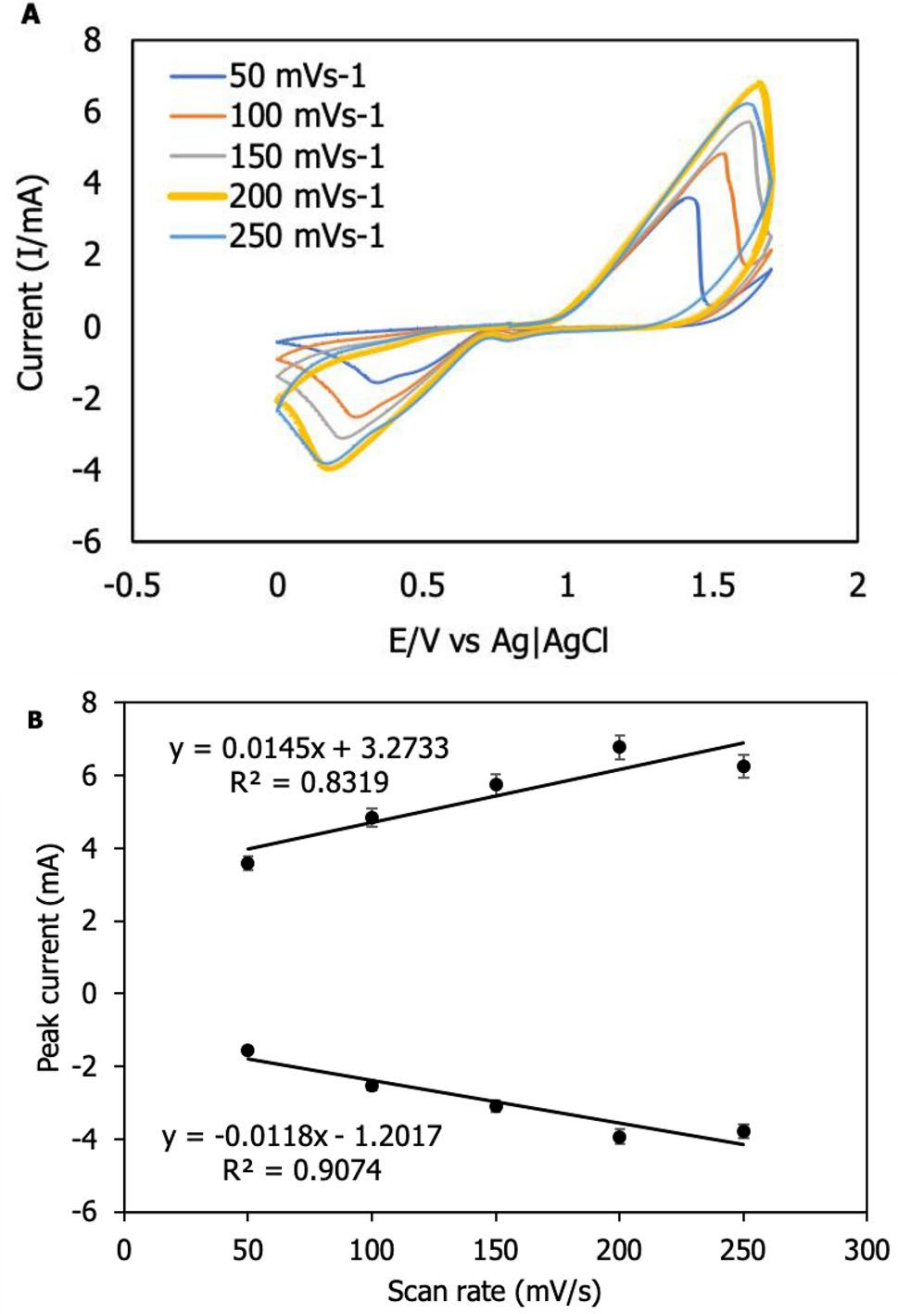
Effect of different scan rates tested on f-MWCNTs/CS/PB/AuE; (**A**) 50–250 mVs^-1^ and (**B**) Linearity of Ipa and Ipc.

The influence of scan rate on f-MWCNTs/CS/PB/AuE was investigated over a range of 50–250 mVs^-1^ (Fig. 6A). Figure 6B shows the oxidation peak (Ipa) and reduction peak (Ipc). According to the figure, Ipa has a linearity of y = 0.0145 ×  + 3.2733 and an R^2^ of 0.8319, whereas Ipc has a linearity of y =  − 0.0118 ×  − 1.2017 and an R^2^ of 0.9074 for f-MWCNTs/CS/PB/AuE. The graph indicates that the influence of the scan rate is proportional to the present reaction. The current responsiveness increased with increasing scan rate from 50 to 200 mVs^-1^. When the scan rate exceeded 250 mVs^-1^, the current's stability deteriorated and the current signal decreased in comparison to 200 mVs^-1^. Due to the system's over-voltage pass, these conditions were not advised for monitoring heavy metal ions. According to Trachioti et al.^39^, the maximum scan rate is 202 mVs^-1^. As a result of the stability of the current response and the greatest current signal obtained, the 200 mVs^-1^ scan rate was chosen as the optimal scan rate for identifying Zn^2+^.

### Effect of accumulation time

**Figure 7:**
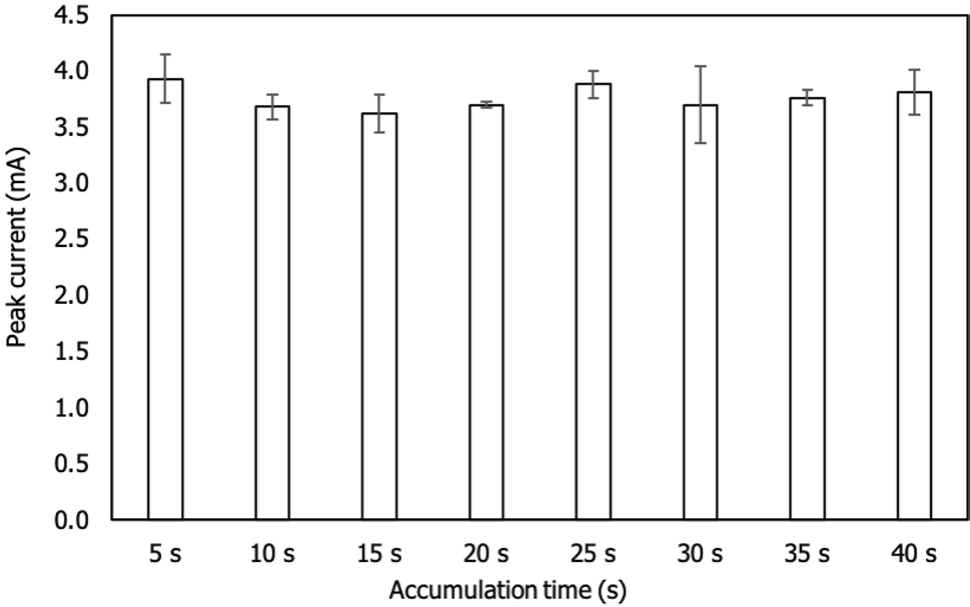
Bar graph of the different accumulation times (5–40 s) on f-MWCNTs/CS/PB/AuE in the presence of 3 ppm Zn^2+^ under 200 mVs^-1^ of scan rate.

The accumulation time was used to determine the target ion's surface saturation^15,19^. The accumulation time was less than one minute, which fulfilled the research objective of developing a rapid sensor. The accumulation time was varied between 5 and 40 s in the presence of Zn^2+^. The effects of accumulation time on Zn^2+^ accumulation is represented in Fig. 7. According to the result, the target ion was originally dispersed throughout the analytical solution before being drawn to the electrode surface by electrostatic interaction^36^. As a result, the current signal was most intense at 5 s, indicating that the target ion was completely covered by the electrode surface. When the accumulation time exceeded 5 s, the current signals appeared unstable. Current signals decreased from 10 to 20 s and then increased to 25 s, then decreased at 30 s, increased again at 35 s and finally decreased somewhat at 40 s. Based on trend, the adsorption equilibrium whereas ion enter in and out between bulk-surface electrode at equal rate was difficult to determine previously described by Behnia et al.^38^. However, this research succeeded to establish optimum time detection of Zn^2+^ was 5 s which possibly the fastest time sensing observed for the past 5 years. The fastest time was discovered at 30 s earlier reported by Roy et al.^36^, they also employed MWCNTs as one of the nanomaterials of the modified electrode for detection of Zn^2+^. Therefore, further optimization employed 5 s as accumulation time.

### Effect of volume ratio

**Figure 8:**
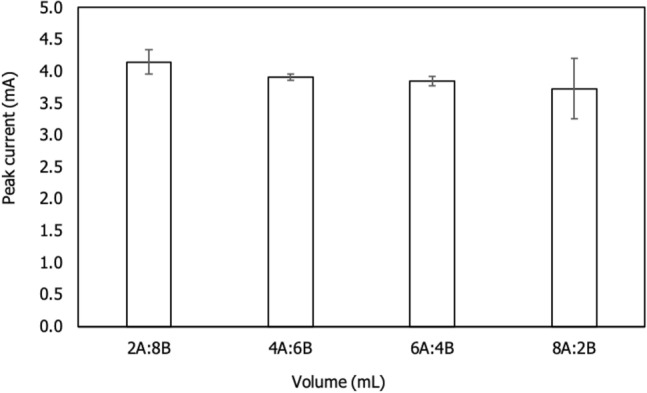
Bar graph was the effects of different analytical volumes (A-analyte, B-buffer) on f-MWCNTs/CS/PB/AuE in the presence of 3 ppm Zn^2+^. The experimental conditions were 0.1 M PBS as supporting electrolyte, 200 mVs^-1^ of scan rate and 10 s of accumulation time.

The total volume of analytical solution was conducted using 10 mL for experiment according to Chaiyo et al.^17^ with some modifications. In this experiment, the volume ration of analyte and buffer assessed for the application on real sample. Based on dilution principle, the concentrations were influenced by volume. Dissolving amount of zinc in solution in buffer solution changes the concentration of zinc as well as ionic strength. Based on our understanding, there is no optimization of volume ratio undertaken yet. Since the mixture of both solutions are under consideration, the ratios were created as 8 mL: 2 mL; 6 mL: 4 mL; 4 mL: 6 mL and 2 mL: 8 mL, respectively. The effects of four various mixing ratio solutions are displayed in Fig. 8. The result showed that 2 mL analyte volume was enough for optimizing of Zn^2+^. Therefore, 2 mL was selected as optimum volume and 8 mL of buffer to generate better current response for the developed sensor.

### Analytical performance

#### Effect of electrode configurations

**Figure 9:**
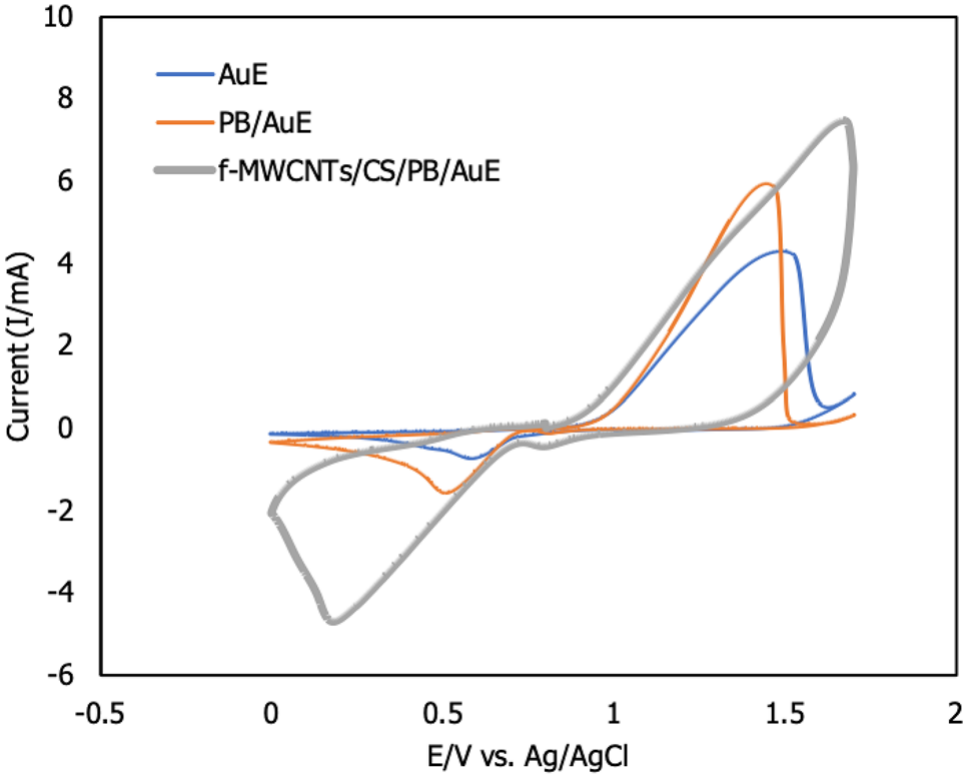
Comparison current signal on different modified electrodes in the presence of 3 ppm Zn^2+^ (*n* = 3).

The CV approach was used to determine the effect of various AuE surface areas on the formation of current signal under optimal parameters in Fig. 9. The CV approach demonstrated the interaction of the target ion with various AuE surfaces. The target ion is oxidized and reduced via gain or release electrons, as indicated by the CV graph. It occurs concurrently throughout a wide potential range of 0.0–1.7 V. As a result of the analyte's prolonged reaction with the modified surface of AuE, strong current signals are achieved. When compared to bare AuE, the performance of the modified electrode (f-MWCNTs/CS/PB/AuE) surface area was significantly increased. The modified AuE's oxidation and reduction current signals were increased incrementally. This finding may contribute to the modified electrode's efficacy in detecting the presence of Zn^2+^ by enhancing both the active area and electron kinetic transfer. Meanwhile, PB was substantially supported by both the bare and modified AuE, which acted as a 'booster' for the current signals. At optimal conditions for detecting the target ion, the combination of f-MWCNTs/CS/PB/AuE provided the maximum current response. This study also demonstrated the use of CS/f-MWCNTs, which has not before been reported in the context of zinc ion detection. The surface-active area of the modified electrodes could be determined using the highest peak current signal observed. Table 2 lists the surfaces of several surface-active areas.

**Table 2:** Surface active area and peak signals of AuE, PB/AuE and modified f-MWCNTs/CS/PB/AuE.

Electrode configuration	Peak signal (mA)		Surface active area (cm^2^)	
	Anodic	Cathodic	Anodic	Cathodic
AuE	4.31	− 0.71	0.03	5.88 × 10^–3^
PB/AuE	5.94	− 1.55	0.04	0.01
f-MWCNTs/CS/PB/AuE	7.46	− 4.67	0.06	0.03

#### Repeatability and reproducibility

**Figure 10:**
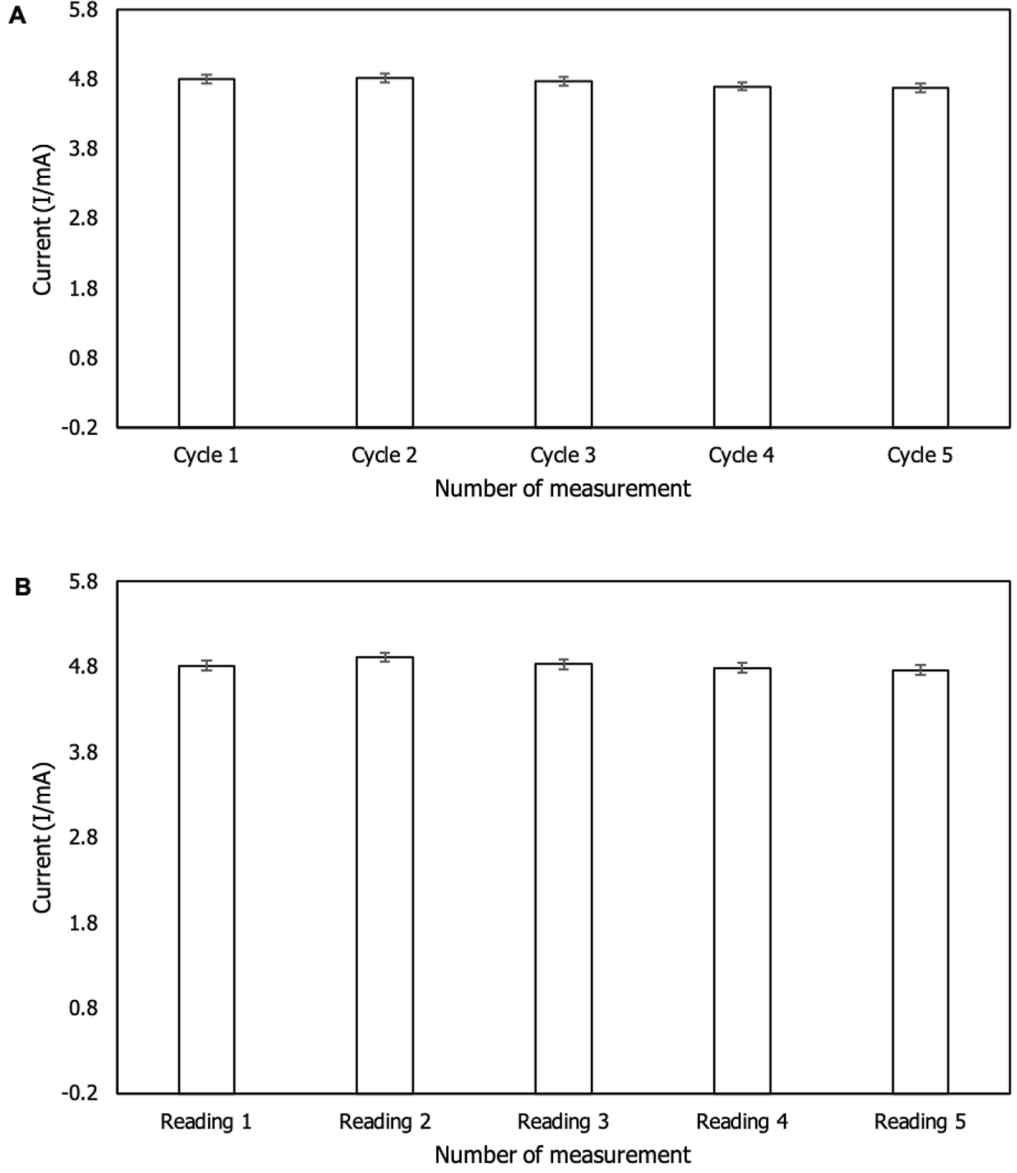
Bar charts of (**A**) repeatability and (**B**) reproducibility tests on f-MWCNTs/CS/PB/AuE in the presence of Zn^2+^ (*n* = 5).

The repeatability of the modified sensor was determined five times using the same procedure and electroanalytical solution. According to Fig. 10A, there was no difference in peak current signals across all measurements, indicating that the modified sensor had excellent repeatability. Meanwhile, five different samples were prepared and examined for repeatability on f-MWCNTs/CS/PB/AuE. The reproducibility result in Fig. 10B indicates that there was no significant difference in the formation of peak signals, indicating that the modified electrode was highly stable when used with different samples tested. The RSD was determined for repeatability and reproducibility by multiplying the mean by the standard deviation and times by 100% (m/SD × 100%). The RSD of repeatability and reproducibility is less than 6% in Table 3, showing that f-MWCNTs/CS/PB/AuE exhibits a high degree of stability in detecting Zn^2+^ in an electroanalytical solution.

**Table 3:** RSD of repeatability and reproducibility of modified sensor.

Characteristic	Mean ± STD	RSD (%)
Repeatability	4.15 ± 0.07	1.73%
Reproducibility	4.10 ± 0.24	5.97%

#### Interfering study

**Figure 11:**
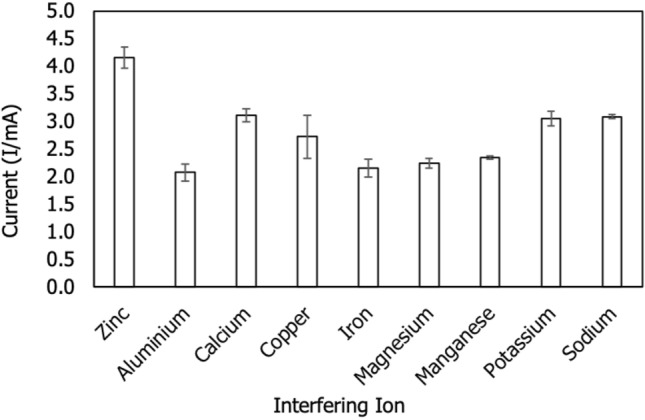
Bar charts of interfering ions for selectivity test of zinc sensor.

The interference investigation was conducted using 10 ml of Zn^2+^ and the corresponding ions. Foreign ions such as aluminium (Al^3+^), calcium (Ca^2+^), copper (Cu^2+^), iron (Fe^2+^), magnesium (Mg^2+^), manganese (Mn^2+^), potassium (K^+^) and sodium (Na^+^) ions were analyzed. These ions were formed by aluminium sulphate (Al_2_(SO_4_)_3_), calcium carbonate (CaCO_3_), copper sulphate (CuSO_4_), iron(II) sulphate (FeSO_4_), manganese(II) sulphate (MnSO_4_), potassium sulphate (K_2_SO_4_) and sodium carbonate (Na_2_CO_3_). The metal ion was introduced at the same concentration to an electroanalytical solution containing 3 ppm zinc ion^19^. 3 ppm of the corresponding interfering ion was generated by diluting in a 2 mL buffer solution containing 3 ppm of Zn^2+^. The current signal was then determined using the DPV approach. The Fig. 11 illustrates the current signals generated by the various ions. The current signals were plotted using the signals with the highest DPV. According to the results, the selectively developed sensor produced the maximum current signal when Zn ion was present. Other ions, on the other hand, produced lower current signals than the Zn ion. We hypothesize that the developed sensor demonstrated a strong selectivity for the Zn ion over other foreign ions. Cu^2+^ is identified as a strong competitor ion by the formation of the Cu–Zn intermetallic complex is not detected^36^. The comparison study between and within groups are analyzed using One-way ANOVA as indicated in Table 4. Based on the table, the signification value for all interfering ions are closed to zero (0) with value of 0.00 indicating no interfering activity occurred.

**Table 4:** The One-Way ANOVA analysis of zinc ion in the presence of interfering ions.

	Sum of squares	df	Mean square	F	Significant value
ANOVA					
**Current**					
Between groups	15.480	8	1.935	68.164	0.00
Within groups	0.511	18	0.028		
Total	15.991	26			

Target ion	Interfering ion	Mean difference	Significant value		
**Post Hoc tests**					
Zinc	Aluminium	0.761	0.00		
	Calcium	2.563	0.00		
	Copper	1.412	0.00		
	Iron	0.825	0.00		
	Magnesium	0.811	0.00		
	Manganese	0.475	0.00		
	Potassium	0.626	0.00		
	Sodium	0.363	0.00		

#### Storage stability

**Figure 12:**
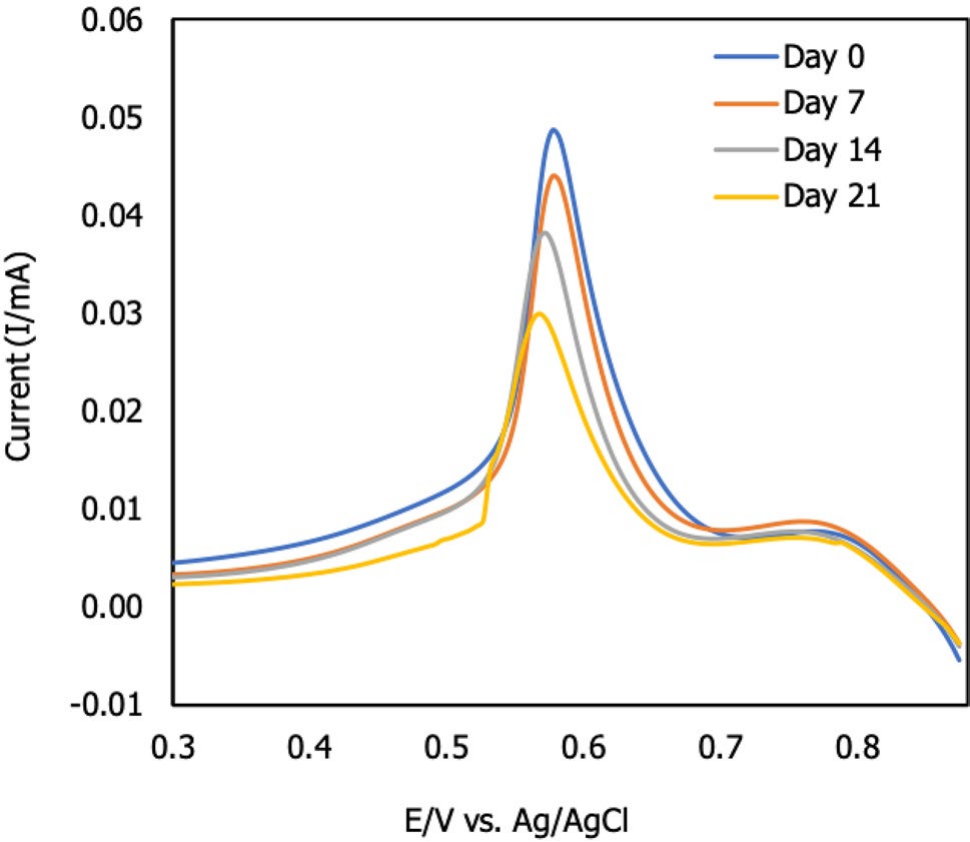
Effect of the storage stability test on modified electrode (f-MWCNTs/CS/PB/AuE) form day 7 to day 21. The modified electrode was measured in the presence of Zn^2+^ (*n* = 3).

The storage stability of f-MWCNTs/CS/PB/AuE was determined for the duration of the modified electrode before current signals were taken. The modified electrode was kept at a temperature of 20 ℃ throughout this experiment. The effect of storage stability in the presence of 3 ppm Zn^2+^ is shown in Fig. 12 using the DPV method. The current intensity indicated that the target ion was attached to the modified surface AuE between 0.3 and 0.85 V. This signal is generated when a target ion releases an electron during the oxidation process. Between 0.3 and 0.85 V, the target ion was suspected of releasing an electron and generated the current signal. According to the results, the current signal decreased as the modified electrode was stored for an extended period of time^40^. The changing current signal fell by 10% from the initial day to 7 days, but decreased by approximately 20% and 24% after that (14 days and 21 days, respectively). This suggested that due to the freshness of the f-MWCNTs/CS/PB/AuE, the term of storage of the modified electrode should be addressed. As a result of this finding, the performance of the modified sensor may degrade if the length of storage is extended.

#### Dynamic range

**Figure 13:**
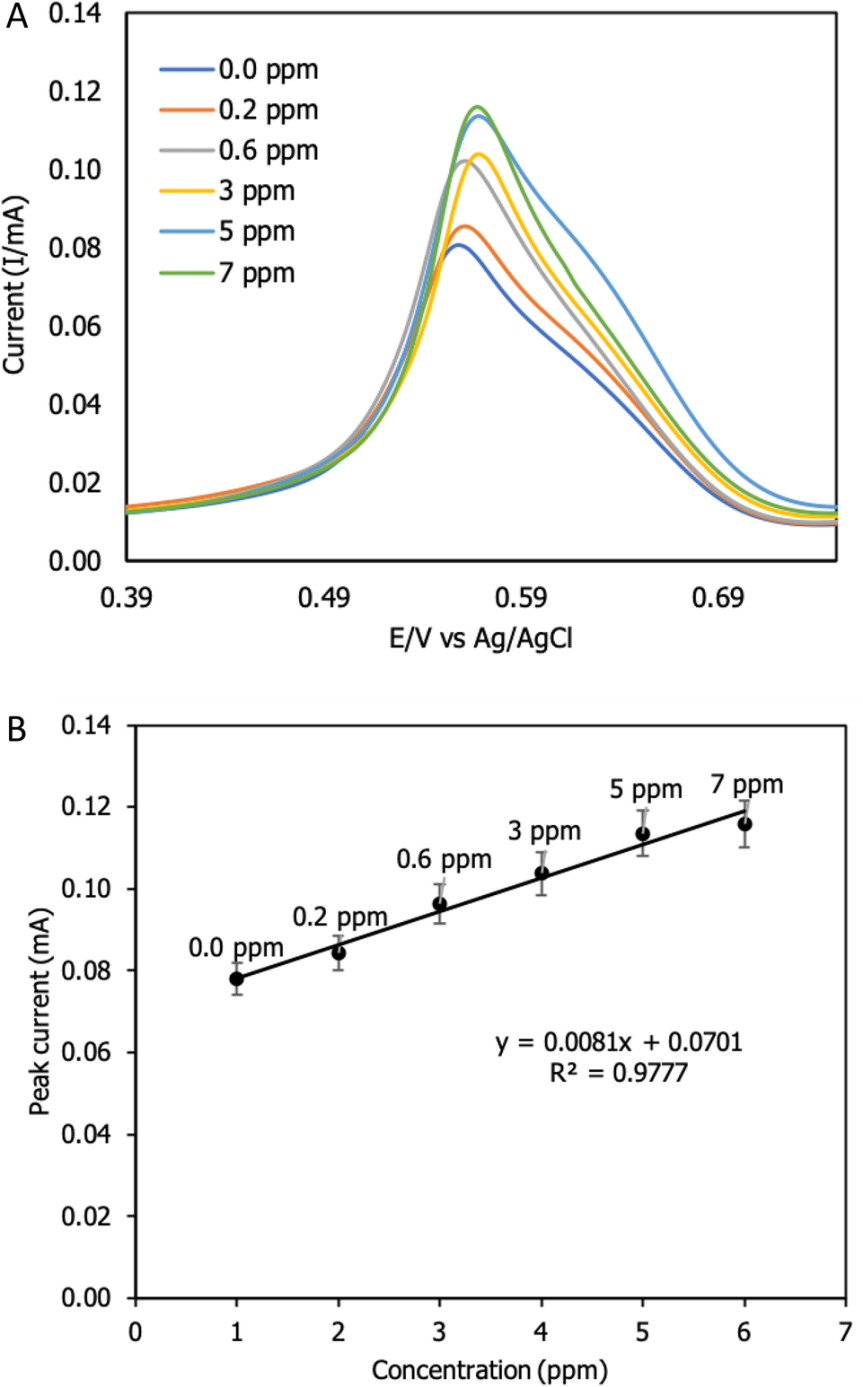
Effect of the different concentrations of Zn^2+^ on modified electrode (f-MWCNTs/CS/PB/AuE): (**A**) DPV analysis of the different concentrations and (**B**) the linearity of the analyte concentration (*n* = 3).

In Fig. 13A, the effect of current signal at various Zn^2+^ concentrations was investigated using the DPV method. The DPV approach was used to determine the linearity of the developed sensor by measuring the current signal at varied concentrations of target ion. The DPV approach has demonstrated that an increase in current signal corresponds to an increase in concentration. However, the potential is changed as increasing the concentrations as same findings by Lee et al.^19^. They found that the Zn^2+^ was not linear over range. We suspected that the modified electrode's large surface area would attract zinc ions as concentration zinc added into electroanalytical solution. Additionally, the developed sensor's performance can be evaluated in terms of the lowest detectable concentration, often known as the limit of detection, or LOD. The LOD provides critical information regarding a nanomaterial's capacity to detect the lowest concentration^41^. The following concentrations were used in this experiment: 0.0 ppm, 0.2 ppm, 0.6 ppm, 3 ppm, 5 ppm, and 7 ppm. According to the Fig. 13B, the graph was linear at y = 0.0081x + 0.0701 with a correlation coefficient of R^2^ = 0.9777. As the concentration increases, the current signals change. Each concentration showed the shifting of the current signal. The impact of nanomaterials demonstrated the new current signal creation associated to various concentration investigated. The limit of detection (LOD) was determined to be 0.017 ppm (2.6 × 10^–7^ M) using formula 3SD/m, while the sensitivity was 0.1099 ppm^-1^ cm^-2^. According to previous research on zinc ion detection in drinking water in Table 5, this study had the second lowest zinc ion detection in drinking water (2.60 × 10^–7^ M) when applied nanocomposites. Trachioti et al.^39^ reported the lowest LOD of 8.4 × 10^–9^ M. These experiments performed the nanocomposite materials on the modified electrode. This research applied multiwalled carbon nanotubes whereas Trachioti et al.^39^ applied graphite. This research proved that nanocomposites have different in mechanical properties and electric conductivity.

**Table 5:** The comparison of the modifications used for determining Zn^2+^ in drinking water.

References	Modification	LOD (mol L^-1^)
This study	Functionalized multiwalled carbon nanotubes/chitosan/Prussian blue/gold electrode (f-MWCNTs/CS/PB/AuE)	2.60 × 10^–7^
^17^	Nafion/ionic liquid/graphene/screen-printed carbon electrode (N/IL/N/G/SPCE)	6.54 × 10^–5^
^36^	Zinc-arginine imprinted electrode	6.53 × 10^–5^
^39^	Tin nanoparticles/graphite screen-printed electrodes (SnNPs/GSPEs)	8.4 × 10^–9^

#### Real sample analysis

The real sample analysis was conducted using DPV method for the modified AuE (f-MWCNTs/CS/PB/AuE) in drinking water was obtained from local grocery shops, Kota Kinabalu, Sabah, Malaysia. The spiking analysis was conducted by comparing the current signals between buffer and drinking water that added at tested concentrations. Three concentrations of Zn^2+^ were examined such as 0.2 ppm, 0.6 ppm and 3 ppm according to the previous three concentrations conducted by Chaiyo et al.^17^ and Behnia et al.^38^. Based on the same articles, Chaiyo et al.^17^ and Trachioti et al.^39^ discovered that their recoveries of produced sensor rates were 106.8% and 90.3–112.5%, respectively. Meanwhile, Munir et al.^16^ obtained the RSD was less than 5%. Despite on the proposed real sample was run to determine the recovery and RSD given in Table 6. Based on the table, we determined that the recovery of the suggested sensor was between 95.78 and 98.96% and the RSD was less than 7%. These data were still reliable for evaluating individual assay as the concentration tested less than 3 ppm and an acceptable range was of 75–120%^42^. Therefore, this produced sensor exhibited the high accuracy by eliminating any possible foreign ions when it applied to evaluate into real sample.

**Table 6:** The recovery value of f-MWCNTs/CS/PB/AuE for detecting Zn^2+^ in drinking water.

Concentration (ppm)	Current signal of concentration added (ppm)	Current signal of concentration found (ppm) Mean ± standard deviation	Recovery (%)	RSD (%)
0.2	8.42 × 10^–2^	8.19 × 10^–2^ ± 3.97 × 10^–3^	95.78	4.84
0.6	9.64 × 10^–2^	9.54 × 10^–2^ ± 5.24 × 10^–3^	98.96	5.49
3	1.03 × 10^–1^	1.00 × 10^–1^ ± 4.13 × 10^–3^	97.08	4.09

## Conclusion

In conclusion, the performance of the modified (f-MWCNTs/CS/PB/AuE) sensor is strongly influenced by the optimization experiment to detect specific zinc ion. The highest signals are exhibited under the ideal settings of optimization experiment. The obtained ideal conditions were PBS (0.1 M, pH 2) supported 5 mM Prussian Blue; scan rate = 200 mVs^-1^; accumulation time = 5 s; and volume 10 mL effectively employed to detect of Zn^2+^ with varying concentrations in the range of 0.2–7 ppm. LOD detected 0.017 ppm (2.6 × 10^–7^ M). There was no interfering ion observed under optimal conditions. The developed sensor exhibited a good RSD value for repeatability and reproducibility with less than 7%. In addition, the developed sensor is employed in real sample analysis; however, there is no contamination of Zn^2+^ ion present in drinking water which is safe to consume. This developed sensor is a sensitive and rapid in monitoring water contamination especially in drinking water.

## Supplementary Information

Supplementary Information.

## Data Availability

All data are included in Supplementary material. All data used in the current research are available from the corresponding author on reasonable request.
