# Supplementary material for: A sensitive and rapid determination of zinc ion (Zn2+) using electrochemical sensor based on f-MWCNTs/CS/PB/AuE in drinking water
Source: Sci Rep. 2022 Nov 3;12:18582. doi: 10.1038/s41598-022-21926-6 (PMC9633590; doi:10.1038/s41598-022-21926-6)
Supplement: Supplementary file 1 — Supplementary Information. [file 41598_2022_21926_MOESM1_ESM.docx]

**Data Availability**

**The CV graph of (0.1 M, pH 2) buffer and 5 mM of redox indicator for detecting Zn^2+^ using unmodified AuE**

1. Phosphate buffer saline
2. Tris-HCl buffer
3. Citrate buffer
4. Ammonium buffer
5. Acetate buffer

**The CV graph of (0.1 M, pH 2) buffer and 5 mM of Prussian blue for detecting Zn^2+^ using modified AuE (f-MWCNTs/CS/PB/AuE)**

**The CV graph of different pH values for detecting Zn^2+^ using modified AuE (f-MWCNTs/CS/PB/AuE)**

1. Modified AuE

**The CV graph of different scan rates for detecting Zn^2+^ using modified AuE (f-MWCNTs/CS/PB/AuE)**

1. Modified AuE

**The CV graph of different accumulation times for detecting Zn^2+^ using modified AuE (f-MWCNTs/CS/PB/AuE)**

1. Modified AuE

**The CV graph of different volume ratios for detecting Zn^2+^ using modified AuE (f-MWCNTs/CS/PB/AuE)**

**The CV graph of different electrode configurations for detecting Zn^2+^.**

1. CV graph of Zn^2+^

Note: CuONPs as nanoparticles were tested in this experiment with overcurrent observed.

**The CV graph of repeatability test for detecting Zn^2+^.**

1. CV graph of Zn^2+^

**The CV graph of reproducibility test for detecting Zn^2+^.**

1. CV graph of Zn^2+^

**The CV graph of interfering study for detecting Zn^2+^.**

1. CV graph of Zn^2+^

**The DPV graph of storage stability for detecting Zn^2+^.**

1. DPV graph of Zn^2+^
